# Predictive Value of Morphological Features in Patients with Autism versus Normal Controls

**DOI:** 10.1007/s10803-012-1554-4

**Published:** 2012-06-06

**Authors:** H. Ozgen, G. S. Hellemann, M. V. de Jonge, F. A. Beemer, H. van Engeland

**Affiliations:** 1 Department of Child and Adolescent Psychiatry, University Medical Center Utrecht, B01.201, PO Box 85500, 3508 GA Utrecht, The Netherlands; 2 Rudolf Magnus Institute of Neuroscience, University Medical Center, Utrecht, The Netherlands; 3 SIStat, Semel Institute for Neuroscience, University of California, Los Angeles, CA USA; 4 Department of Medical Genetics, University Medical Center, Utrecht, The Netherlands

**Keywords:** Autism, Common variant, Endophenotype, Morphology, Minor anomaly, Predictive value, ROC, Recursive partitioning

## Abstract

We investigated the predictive power of morphological features in 224 autistic patients and 224 matched-pairs controls. To assess the relationship between the morphological features and autism, we used the receiver operator curves (ROC). In addition, we used recursive partitioning (RP) to determine a specific pattern of abnormalities that is characteristic for the difference between autistic children and typically developing controls. The present findings showed that morphological features are significantly increased in patients with autism. Using ROC and RP, some of the morphological measures also led to strong predictive accuracy. Facial asymmetry, multiple hair whorls and prominent forehead significantly differentiated patients with autism from controls. Future research on multivariable risk prediction models may benefit from the use of morphological features.

**Electronic supplementary material:**

The online version of this article (doi:10.1007/s10803-012-1554-4) contains supplementary material, which is available to authorized users.

## Introduction

Autism Spectrum Disorder (ASD) represents a set of chronic and severe neurodevelopmental disorders of childhood characterized by qualitative impairments in social interaction and communication skills, accompanied by repetitive and stereotyped behaviors and interests. These symptoms manifest in the first 3 years of age and show a lifelong persistence (APA 1994). The prevalence of ASD is estimated to be approximately 1 in 150, with a male to female ratio of 4:1 (Chakrabarti and Fombonne 2005; Veenstra-VanderWeele and Cook 2004). A more recent study even indicated a prevalence rate of 1 in 100 (Baird et al. 2006). Family and twin studies have shown that ASD has a strong heritable component, but the pattern of inheritance is not straightforward and is likely to involve complex interactions between multiple genes and possibly environmental insults (Zhao et al. 2007).

Despite the significant heritability, identifying specific causal relationships has been daunting due to genetic complexity and phenotypic variation (Geschwind 2008). Such heterogeneity in autism has led researchers to seek for reliable diagnostic tools to identify genetically more homogeneous subgroups to reduce the complexity of the task of identifying influential genes. Most studies have used variations in functional or behavioral measures as the basis for the stratification (Geschwind 2008; Sebat et al. 2007; Klin et al. 2007), whereas others assessed the contribution of de novo copy number variants (CNVs) to ASD in a unique large sample, namely, the Simon simplex collection (Fischbach and Lord 2010; Levy et al. 2011; State and Levitt 2011).

The study of head circumference and other morphological characteristics has appeared in more recent autism research as a way of stratifying more homogenous subgroups. Excessive head growth found in the first year of life, in children later diagnosed with autism, has been one of the most promising quantitative traits (Miles et al. 2000; Sacco et al. 2007). As to other morphological characteristics, an excess of minor physical anomalies (MPAs) in autistic individuals received specific attention (Steg and Rapoport 1975; Gualtieri et al. 1982; Hardan et al. 2006; Miles et al. 2008; Ozgen et al. 2010a, b). Recently, the largest study to date comparing morphological features in 224 autistic patients and 224 matched-pairs controls, showed that the morphological abnormalities were significantly more prevalent in patients with autism than in the normal control group and 48 morphological features distinguished patients from controls (Ozgen et al. 2010a, b). However, although there is now robust evidence for the association between morphological features and autism no studies, to date, have directly assessed the utility of various morphological indices in characterizing ASD patients. The utility of a test is defined by its sensitivity and specificity. The specificity of an index reflects the likelihood that an individual belonging to a comparison group is identified as not abnormal on the index (a true negative), while the sensitivity of an index reflects the likelihood that an individual that should be classified as belonging to the abnormal group is identified correctly (a true positive). The sensitivity and specificity of an index for differentiating a diagnostic group from a comparison group are always a trade-off, i.e., if one chooses a cut-off point that increases sensitivity, the specificity will be decreased, and vice versa. The receiver operator characteristic (ROC) analysis is used to characterize sensitivity and specificity across the full range of potential cut-off points. As we are interested in the overall performance of the measures we present the results in the form of the ROC (Hanley and McNeil 1982; Wickens 2002).

However, to date, there have been few systematic attempts to use the aggregated information of these multiple markers of ASD. As part of a larger effort to investigate morphological features in ASD, we performed additional analysis on the data of our recently published study in 224 children with ASD and 224 matched-pairs controls and focused specifically on the potential value of exploring these features in ASD samples and attempted to determine the value of morphological features in distinguishing ASD patients from normal controls. In this study, we use ROC analysis to establish that aggregate measures of morphological abnormalities offer a large amount of information regarding ASD, and then use recursive partitioning, a data mining technique, to establish and validate a parsimonious clinical decision rule.

## Materials and Methods

### Study Participants

Between February 2006 and March 2007, we examined all consecutive patients attending the Department of Child and Adolescent Psychiatry (CAP) of the University Medical Center in Utrecht (UMCUtrecht), the Netherlands. This hospital is a tertiary referral center and provides health services to a wide range of patients from mainly the centre and south of the Netherlands. The CAP runs a clinic specifically dedicated to assessment of children suspected with autism and psychosis. During the study period, patients and/or their caregivers were invited in writing to participate both to psychological assessments as to a physical examination.

Patients with ASD were included if the following criteria were met: (1) a DSM-IV diagnosis of ASD; (2) absence of any known syndrome and (3) absence of mental retardation; namely IQ > 70. Consensus diagnoses were made for each case, based on a developmental history, behavioural observation, medical examination, and all information in the clinical file. In addition, the accepted standard for autism diagnosis, the Autism Diagnostic Interview-Revised (ADI-R) (Lord et al. 2004) was administered in 168 patients to confirm their previously determined clinical diagnosis of autism.

Ethnicity was registered because it can influence the external phenotype (McGrath et al. 2002; Merks et al. 2008). Ethnicity was classified as Caucasian or non-Caucasian. Laboratory testing included routine conventional karyotyping, DNA for Fragile X and urine metabolic screen. In addition, array comparative genomic hybridization (CGH) and single nucleotide polymorphism (SNP) arrays were requested from a clinical geneticist, when needed.

The study procedures were approved by the medical ethics committee (METC) of the University Medical Center, Utrecht, the Netherlands. Patients entered the study only after written informed consent was obtained from themselves and/or their parents.

A total of 1,007 typically developing schoolchildren were used as controls; they were examined and analyzed in an identical way by the same primary investigator. Additional details on the study design and data collection results of the control cohort are available elsewhere (Merks et al. 2006).

### Terminology and Classification of Morphological Features

A hierarchical tree was built, comprising 29 major anatomical areas, subdivided into 98 different structures, and containing 683 standardized morphological abnormalities. The morphological abnormalities were classified according to their (presumed) pathogenesis, and subdivided into (a) Major abnormalities, caused by abnormal development; and (b) Minor variants. The minor variants can be subdivided into two categories, based on their prevalence in the normal population (Merks et al. 2008) (Supp. Fig. 1).

### Morphological Examination (Qualitative and Quantitative Measures)

All patients and controls were carefully examined in an identical way by the same trained examiner. The clinical examination consisted of standard morphological measurements and comprised a broad range of qualitative and quantitative physical measurements. All items in the Waldrop-scale (Waldrop et al. 1968) were included in this list and a clear differentiation between major abnormalities, minor anomalies and common variants were introduced. It should be emphasized that the distinction between major and minor anomalies is pragmatic, and only defined by the effect on the child. Techniques and standards of measurement were adapted from the studies by Aase (1990) and Hall et al. (2007). Height, weight, head circumference, inner and outer canthal distance, ear length, hand length and palm length were measured and shoe size was converted into foot length. No specific equipment other than a ruler and measuring tape was used. Auscultation of the heart, abdominal palpation, examination of internal organs and of the external genitalia was not performed. Body mass index (BMI) is formulated as weight (kg)/height (m^2^) and interpreted with the reference of Van Buuren (2004). Palpebral fissure length (PFL) is defined as the distance between the inner and outer canthus of one eye (Hall et al. 2007). Reliability studies were conducted using a second observer (clinical geneticist/pediatrician) who examined 30 patients (9 %) of the ASD group, blind to the patients’ diagnosis and to the results of morphological assessment of the first examiner. This resulted in a kappa score of 0.81 (Cohen 1960). For the controls, 111 children (11 %) were examined by a second observer resulting in a kappa score of 0.85. Assessors were blinded to family status and to any previous diagnosis at the time of the assessment.

### Statistical Analyses

To best characterize the pattern of any potential dysmorphogenesis in patients with autism, we compared each of the quantitative and qualitative items in carefully selected matched pairs. To reduce the influence of ethnic variability in the matched-pairs, analyses were restricted to Caucasian patients and controls. We used procedures in SPSS to match as many patients with autism as possible to controls based on sex and age (±2 years). When more than 1 control was available for matching to a case, the final match was randomly selected from the pool. Patients with no matching comparison subject were excluded from the analysis.

To determine the overall strength the relationship between morphological features and autism we first created four indices that describe the degree of dysmorphgenesis: a count of common variants, a count of minor anomalies, a count of major abnormalities and the overall count. To determine the strength of the relationship between these indices and autism we determined the receiver operator curve (ROC) and the area under this ROC for each of them. In addition to that we also generated a decision rule based on equally maximizing both sensitivity and specificity. For specific applications other tradeoffs between sensitivity and specificity might be better, e.g. tests used for screening tend to favour sensitivity, while diagnostic tests favour specificity (Ozgen et al. 2010a, b).

In addition, to determine whether if specific patterns of abnormalities are needed to characterize the difference between autistic children and healthy controls, we used recursive partitioning (RP) to determine what decision tree is the most parsimonious representation of the information. This decision tree is represents an alternative to the dysmorphia indices, and offers the opportunity to evaluate their performance at modeling the difference between autistic children and controls with a empirically derived parsimonious decision rule. We included all of the measurements that we had available as potential predictors in the analysis. RP examines all predictors and identifies a hierarchy of variables that are, in succession, most predictive of the subsequent diagnosis. RP allows researchers to include any number of predictor variables in their analysis, regardless of the number of observations, even including the special case of analyzing a dataset that has more variables than observations. Zhang and Singer (1999) present a comprehensive overview of RP methodology. These features have made RP a popular technique for genetics research, where large numbers of variables and relatively small sample sizes are common. For example, Batliwalla et al. (2005), using RP, examined the expression of 12,509 genes on a sample with 19 affected patients and 19 controls. RP is also becoming more prevalent in the examination of treatment outcomes in medical fields other than genetics. Dennison et al. (2007) used RP to identify risk profiles in patients with Parkinson’s disease who needed physical therapy, and Wang et al. (2006) used RP to identify which pediatric patients and their caretakers are least likely to comply with physicians instructions. In general, RP results have been used to identify variables that merit increased attention in subsequent research, to suggest treatment guidelines, and to identify potential risk factors in a wide range of fields. Here we attempt to determine which—if any—specific physical abnormalities or combinations of abnormalities are indicative of ASD.

## Results

### Demographics

The summaries of data and further information are in supporting information (SI) (Suppl Tables 1a and 1b and 2). A total of 442 patients were invited to participate; of these, 421 agreed to undergo extensive morphological assessments (95.2 %). The mean age at examination of the patients was 9.7 years (range 3–18 years), compared with 10.4 years (range 8–14 years) for controls. The male/female ratio was 4.19 in patients and 0.93 in controls. Because of the large age and gender differences between autistics and controls, analyses were performed on matched pairs. There were no significant group differences between case and controls with regard to their socioeconomic background. Supp. Table 1 shows the baseline characteristics for the entire sample and the matched sample. Among 421 cases, 32 subjects were excluded from the analysis as being non-Caucasian, 31 were excluded as not fulfilling ASD criteria. Further, 28 children were excluded from the autistic group due to a diagnosis of a known syndrome or chromosomal abnormality (Supp. Table 1b).

After matching on age and gender, 224 patients with autism and 224 controls were available for analysis. Of the 224 matched pairs, 186 (83 %) were male, and 38 (17 %) were female. The mean (SD) age of patients was 10.6 (2.5) years and the mean age of controls was 10.6 (1.4) years.

Reliability studies were conducted using a second observer (FAB, clinical geneticist/pediatrician) who examined 30 patients (9 %) of the ASD group, blind to the patients’ diagnosis and to the results of morphological assessment of the first examiner. This resulted in a kappa score of 0.81 (Cohen 1960). For the controls, 111 children (11 %) were examined by a second observer (clinical geneticist/pediatrician) resulting in a kappa score of 0.85. Assessors were blinded to family status and to any previous diagnosis at the time of the assessment.

### ROC Curve Analysis

**Fig. 1:**
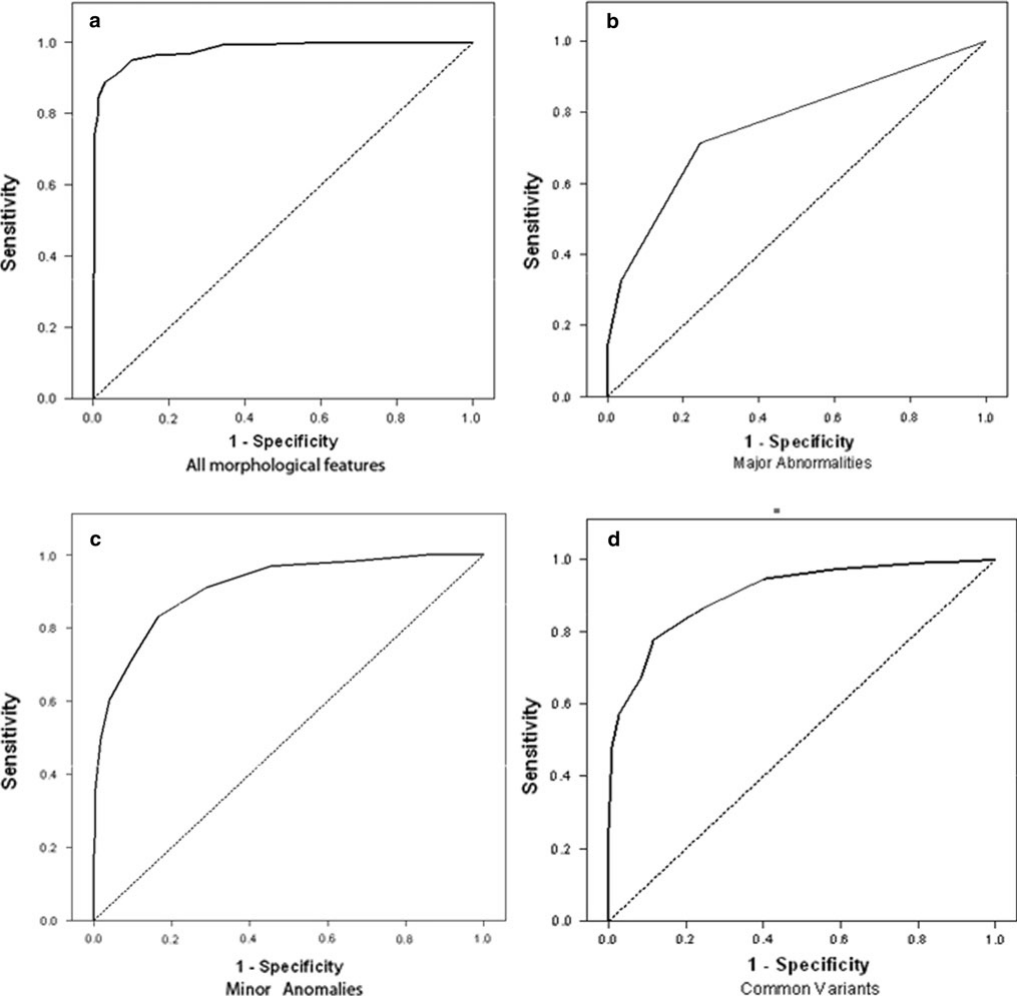
Receiver operator characteristic (ROC) plots from comparisons of matched patients with autism and normal comparison subjects on the. **a** Whole scale, **b** major abnormalities, **c** minor anomalies and **d** common variants in the matched sample of autistics versus controls

Receiver operating characteristic curves are displayed in Fig. 1.

#### Whole Scale of Morphological Features

There were large differences between patients and controls on the whole scale. Controls showed an average of 9.5 abnormalities, with the minimum being 2 and the maximum being 26. In contrast to this, children in the ASD group showed an average of 23.6 abnormalities, with a minimum of 9 and a maximum of 48. The difference is highly significant (*p* < .001) Based on the ROC analysis (Fig. 1a) the best overall decision rule is at ≥16 abnormalities. This yields an overall misclassification rate in this sample of 7 %, with a sensitivity of 0.89 and a specificity 0.97. This decision rule explains a significant amount of the variability in the data, with an area under the curve of 0.97 (*p* < .001).

#### Major Abnormalities

There were large differences between patients and controls on the major abnormalities subscale. Controls showed an average of 0.3 abnormalities, with the minimum being 0 and the maximum being 2. In contrast, children in the ASD spectrum showed an average of 1.3 abnormalities, with a minimum of 0 and a maximum of 8. The difference is highly significant (*p* < .001). Based on the ROC analysis (Fig. 1b) the best overall decision rule is at ≥1 major abnormalities. This yields an overall misclassification rate in this sample of 27 %, with a sensitivity of 0.71 and a specificity 0.75. This decision rule explains a significant amount of the variability in the data, with an area under the curve of 0.76 (*p* < .001).

#### Minor Anomalies

We also found large differences between patients and controls on the minor anomalies subscale. Controls showed an average of 5.7 abnormalities, with the minimum being 2 and the maximum being 13. In contrast, children in the ASD spectrum showed an average of 10.6 abnormalities, with a minimum of 4 and a maximum of 22. The difference is highly significant (*p* < .001). Based on the ROC analysis (Fig. 1c) the best overall decision rule is at ≥8 minor abnormalities. This yields an overall misclassification rate in this sample of 17 %, with a sensitivity of 0.83 and a specificity 0.83. This decision rule explains a significant amount of the variability in the data, with an area under the curve of 0.91 (*p* < .001).

#### Common Variants

A similar pattern of large differences between patients and controls was found on the common variants subscale: Controls showed an average of 3.2 common variants, with the minimum being 0 and the maximum being 10. In contrast, children in the ASD spectrum showed an average of 8.3 abnormalities, with a minimum of 0 and a maximum of 19. The difference is highly significant (*p* < .001). Based on the ROC analysis (Fig. 1d) the best overall decision rule is at ≥6 common variants. This yields an overall misclassification rate in this sample of 17 %, with a sensitivity of 0.78 and a specificity 0.88. This decision rule explains a significant amount of the variability in the data, with an area under the curve of 0.90 (*p* < .001).

### Recursive Partitioning

#### Correlates of Autism

**Fig. 2:**
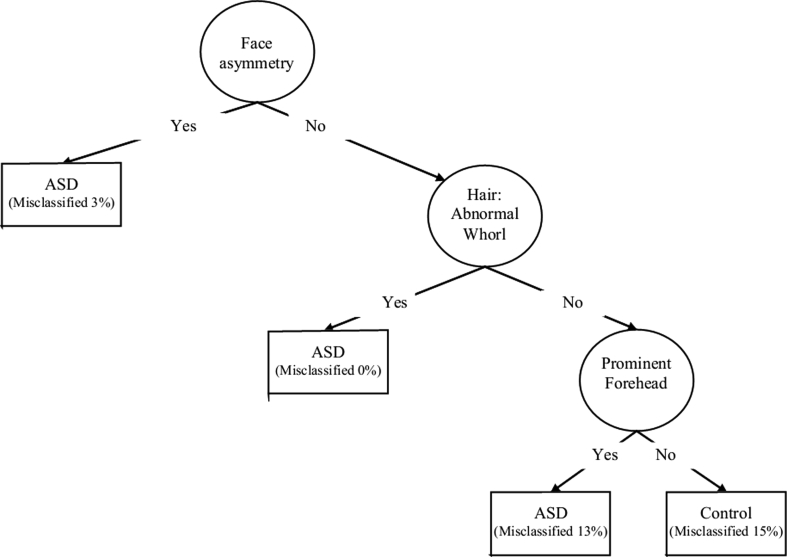
Final decision tree depicting the prediction of morphological features

The tree in Fig. 2 shows the most parsimonious set of variables that relate to autism status in our sample. The specificity of this decision rule is 0.83 and the sensitivity is 0.96. The overall misclassification rate in this sample is 10 %. Using 20-fold cross-classification to determine the expected misclassification rate in the population yields an estimate of also 10 %, suggesting that this tree is very stable and not a consequence of characteristics of this specific sample. The cluster of abnormalities defined by face asymmetry, hair: abnormal whorl (not-frontal) and prominent forehead is highly indicative of autism in this sample, and most likely also reliably separates autistic patients from typically developing controls in the population.

#### Diagnosis Within Autism Subgroups

ASD comprises autistic disorder, Asperger syndrome and pervasive developmental disorder- not otherwise specified. Using RP we also tried to determine which physical abnormalities are associated with different diagnoses within the autism spectrum. None of the physical abnormalities was associated at more than chance level with a specific diagnosis. In addition, a subset of 168 patients had the additional information available in the form of detailed Autism Diagnostic Interview-Revised (ADI-R) (Lord et al. 2004) data. Similarly, we used RP with using ANOVA loss function to accommodate continuous variables to examine the relationship between physical abnormalities and the three subscales Social, Communicative, and Repetitive behavior of the ADI-R and age of first words and age of first phrases and the factor scores based on (Cuccaro et al. 2003). Again, the association did not exceed the chance level.

## Discussion

In this study we further analyzed the findings of our previously published study comparing morphological features in a large cohort of autistic children and matched-paired controls. Here, we sought to determine the sensitivity and specificity indices based on these morphological features in ASD, in comparison with normal control subjects (Fig. 1a–d). This is the first large scale study to explicitly examine the predictive value of morphological features in patients with autism. Several interesting findings emerged from this study.

ROC analysis indicated that the higher prevalence of dysmorphic features in ASD as measured on the whole scale of the morphological features as well as well as on the subscales of major and minor abnormalities is a powerful predictor, which showed extraordinarily high specificity and sensitivity for detecting ASD.

By employing recursive partitioning, we have identified specific morphological features whose expression may be useful diagnostically in discriminating ASD and control subjects, as shown in Fig. 2. These new findings provide a proof of principle and may have the potential to become the basis for the development of diagnostic or prognostic tests. Of the morphological measures used in this investigation, asymmetry of the face, multiple hair whorls and prominent forehead were most specific to ASD patients. The topographical distribution of dysmorphology in our study is consistent with the literature. Other clinical researchers also proposed to use dysmorphology as a tool to delineate heterogeneity in autism by looking for biologically based phenotypes found in consistent proportions of ASD individuals (Miles et al. 2008).

Asymmetry of the face in ASD has been recently documented in a recent 3D morphology study (Hammond et al. 2008). However, asymmetry of the face has also been found in patients with schizophrenia (Weinberg et al. 2007). As we confirmed the higher rate of morphological features in autistic patients as compared to normal controls, we are faced with new challenging questions.

First, why do autistic patients have higher rates of morphological features? Apparently, a common genetic vulnerability for developing autism is reflected in morphological features (Rzhetsky et al. 2007). Several developmental genes have recently been identified that play a paramount role in shaping body structures. Moreover, new insights into craniofacial morphogenesis have indicated that a rapidly increasing number of genes are known to regulate cerebrocraniofacial development (LaMantia 1999). It can be speculated that the genes that determine the craniofacial morphology overlap with candidate genes for autistic disorders. Alternatively, the observed correlations between specific morphological features and autism might be the effects of temporal exposure of different anlage to as yet undefined factors that impact growth and development.

Although our findings indicated that morphological features could have a predictive value for the diagnosis of autism, an intriguing question concerns their specificity concerning ASD subtypes and other neurodevelopmental psychiatric disorders. Although we predicted that morphological features should be able to classify autistic patients into different subgroups, our data did not support this hypothesis. One reason could be that patients were not selected on the basis of these different subtypes and therefore that not all subtypes were equally prevalent in our study population. Additionally, characterization based on DSM IV subtypes may not relate to underlying differences in etiology. Larger studies that are designed specifically to assess morphological features in different autistic subpopulations are needed to specifically investigate this issue. Another question concerns whether morphological features found in autism differ from those found in other disorders. In a recent meta-analysis, a higher prevalence of morphological features was also established in schizophrenia (Weinberg et al. 2007). Do morphological features seen in autism have a different etiology than those in schizophrenia, or do disorders associated with morphological features share a common etiological basis with schizophrenia and autism? Some evidence for such an overlap comes from the observation that individuals with ASD may also be at greater risk for developing schizophrenia (Murphy and Owen 1996; Esterberg et al. 2008). Emerging studies have described the possible links between the two disorders by means of the genetic overlapping (Carroll and Owen 2009; Gejman et al. 2011). Findings indicating overlapping markers could provide important clues regarding the underlying genetic bases of these disorders.

This study had some limitations that should be borne in mind when interpreting the results of this study. There have been several approaches to delineate more homogenous subgroups within autism, and those attempts have also been influenced by diagnostic bias as shown by the Simons Simplex Collection analysis. A huge limitation originates from the complex behavioral phenotype of ASDs. Due to the multifactorial nature of the disease, each individual aberration has a modest effect, and the gene–gene interaction and/or gene-environment interaction may attribute to the observed phenotype. Currently, we do not have a coherent understanding of the relationship of genotype and phenotype in ASDs (State and Levitt 2011). Moreover, robust diagnostic specificity is often lacking for endophenotypes and reflects the fact that different disorders may share genes, and also share partially overlapping neural substrate dysfunction and clinical features (Braff et al. 2007).

Second, as morphologic examination requires in-person examination, it is generally not possible for the raters to be blind to diagnosis. Although we made every attempt to ensure that the assessments were carried out blindly to diagnosis, we acknowledge that blinding may not have been complete. However, to prevent observer bias, 11 % of controls and 10 % of patients were scored independently by two observers, resulting in very high kappa scores. Additionally there were no prior hypotheses as of which morphological abnormalities should be associated with autism, and the finding that some morphological abnormalities were not associated at all with the diagnosis, or were even more frequent in controls than in patients suggests that there was no general rater bias.

Third, we used typically developing children as a comparison group in this study. Future research may extend the findings of this study by investigating non-ASD neurodevelopmental disorders such as schizophrenia, ADHD and bipolar disorders.

Fourth, in order to have a homogenous sample we limited our study population to Caucasian patients and controls; because ethnicity can influence the prevalence of morphological abnormalities. Future studies are needed to establish similar norms for other ethnic groups. Likewise, we restricted ourselves to non-mentally retarded, high functioning ASD patients. Therefore, we cannot generalize our findings to mentally retarded ASD patients.

Despite these limitations, the present study provides evidence that morphological features are significantly increased in the patients with autism and that some unknown prenatal biological mechanism is likely responsible for producing these anomalies which may yield further knowledge about the developmental origins of the disease. If independently replicated, the findings have potential utility for early detection of ASD.

## Electronic supplementary material

Below is the link to the electronic supplementary material.

Supplementary material 1 (JPG 188 kb)

Supplementary material 2 (JPG 124 kb)

Supplementary material 3 (DOC 206 kb)

Supplementary material 4 (DOC 28 kb)
