# Supplementary figures and images for: Predictive Value of Morphological Features in Patients with Autism versus Normal Controls
Source: J Autism Dev Disord. 2012 Jun 6;43(1):147–55. doi: 10.1007/s10803-012-1554-4 (PMC3536966; doi:10.1007/s10803-012-1554-4)

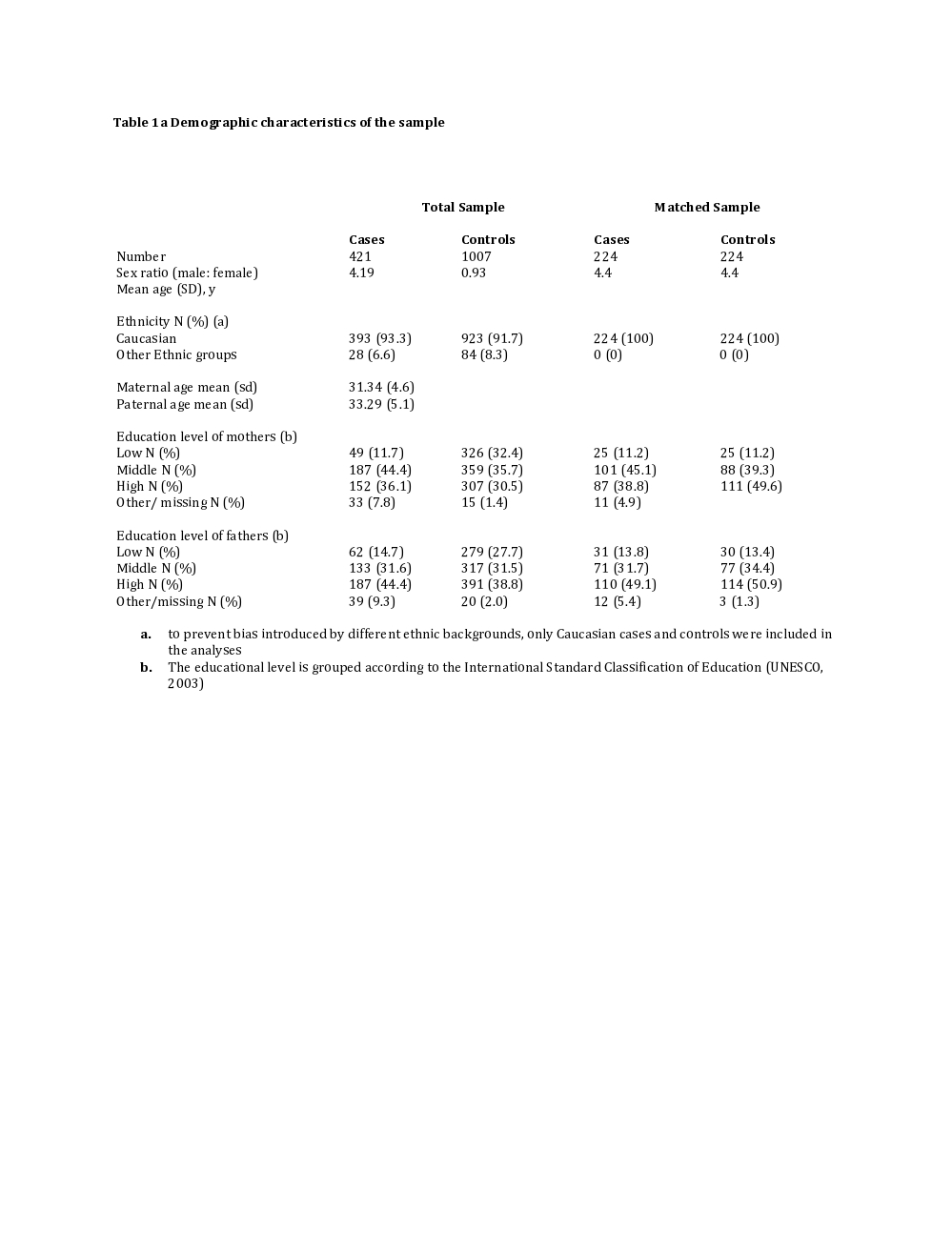

Supplement: Supplementary file 1 — Supplementary material 1 (JPG 188 kb) [file 10803_2012_1554_MOESM1_ESM.jpg]

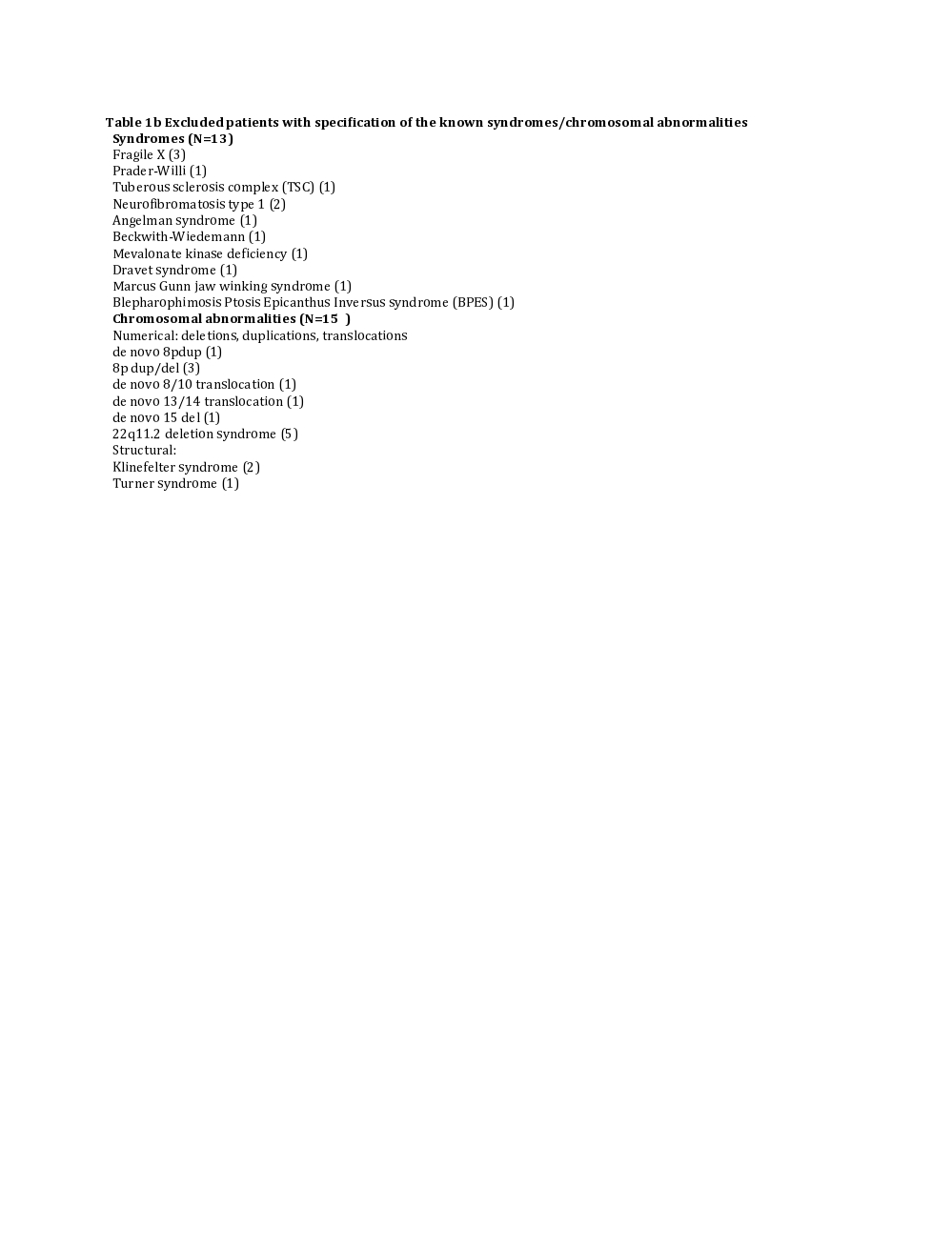

Supplement: Supplementary file 2 — Supplementary material 2 (JPG 124 kb) [file 10803_2012_1554_MOESM2_ESM.jpg]
