# Supplementary material for: Predictive Value of Morphological Features in Patients with Autism versus Normal Controls
Source: J Autism Dev Disord. 2012 Jun 6;43(1):147–55. doi: 10.1007/s10803-012-1554-4 (PMC3536966; doi:10.1007/s10803-012-1554-4)
Supplement: Supplementary file 3 — Supplementary material 3 (DOC 206 kb) [file 10803_2012_1554_MOESM3_ESM.doc]

Supp.Table2. Morphological features occurring significantly more frequent in children with autism vs controls

Matched-sample

| Morphological feature | Controls  (n=224) | Autism  (n=224) | OR  (95% CI) | P | Type of abnormality |
| --- | --- | --- | --- | --- | --- |
| §Brachycephaly | 0 | 18 | ∞ (1.5-∞) | 0.001 | Minor anomaly |
| §#Face asymmetry | 4 | 113 | 27.6 (19.3-73.9) | <0.001 | Minor anomaly |
| §Face coarse | 2 | 27 | 12.9 (3.2-52.5) | <0.001 | Minor anomaly |
| §Forehead prominent | 6 | 53 | 8.2 (3.5-19) | <0.001 | Minor anomaly |
| §Eyes assymetry | 0 | 15 | 13.7 (2-95.1) | 0.001 | Minor anomaly |
| §Eyes deeply set | 9 | 42 | 6.4 (2.7-15) | <0.001 | Minor anomaly |
| §Prominent premaxilla | 4 | 30 | 7.3 (2.6-20.6) | 0.001 | Minor anomaly |
| §Mounth asymmetry | 0 | 23 | ∞ (2.3-∞) | <0.001 | Minor anomaly |
| Macrostomia | 0 | 14 | ∞ (0.9-∞) | <0.001 | Minor anomaly |
| Microstomia | 2 | 15 | 14 (1.8-106) | 0.022 | Minor anomaly |
| §Short philtrum | 5 | 35 | 6.9 (2.7-17.4) | <0.001 | Minor anomaly |
| Upper lip cupid bow | 5 | 25 | 5.9 (2.1-16.7) | 0.032 | Minor anomaly |
| §#Palate high/narrow | 17 | 83 | 5.7 (3.2-10) | <0.001 | Minor anomaly |
| §Long uvula | 3 | 23 | 7.5 (2.3-24.4) |  | Minor anomaly |
| §Macrodontia/teeth asymmetry/abnormally shaped teeth | 3 | 33 | 7.8 (2.8-21.8) | <0.001 | Minor anomaly |
| §Ear low-set | 1 | 20 | 18.3 (2.7-124) | 0.004 | Minor anomaly |
| §Ear posteriorly rotated | 5 | 33 | 10 (3.1-32.2) | <0.001 | Minor anomaly |
| Ear lobe crease | 0 | 12 | ∞ (0.6-∞) | 0.035 | Minor anomaly |
| §Toes syndactyly | 2 | 61 | 13.9 (3.4-56.2) | <0.001 | Minor anomaly |
| §Halluces valgus | 4 | 29 | 9.1 (2.8-29.3) | <0.001 | Minor anomaly |
| §Hypermobile/hyperlax joints | 21 | 74 | 5.4 (2.9-9.9) | <0.001 | Minor anomaly |
| Multiple nevi | 9 | 28 | 3.2 (1.5-6.8) | 0.031 | Minor anomaly |
| Hypo-/depigmentation patches | 4 | 18 | 5.5 (1.6-18.5) | 0.033 | Minor anomaly |
| §Face expression less/dull | 0 | 15 | ∞ (1-∞) | 0.001 | Major abnormality |
| §Open mouth appearance | 0 | 18 | ∞ (1.5-∞) | <0.001 | Major abnormality |
| § ^a^ Abnormal whorl (non-frontal) | 0 | 87 | ∞ (8.4-∞) | <0.001 | Common variant |
| §Full cheeks | 5 | 31 | 9.3 (2.9-30) | <0.001 | Common variant |
| §Periorbital fullness | 8 | 49 | 7.7 (3.3-17.9) | <0.001 | Common variant |
| §Eyebrows arched | 1 | 37 | 33.7 (5-∞) | <0.001 | Common variant |
| §Large nose | 4 | 29 | 7 (2.5-19.9) | 0.002 | Common variant |
| §Nose bridge prominent | 28 | 66 | 3.5 (1.9-6.2) | <0.001 | Common variant |
| §Nose concave | 4 | 27 | 6.6 (2.3-18.6) | 0.006 | Common variant |
| Lower lip full | 31 | 65 | 2.6 (1.6-4,4) | 0.001 | Common variant |
| §Upper lip thin | 21 | 60 | 3.4 (1.9-5.9) | <0.001 | Common variant |
| §Prominent philtrum | 5 | 30 | 5.9 (2.3-15) | 0.004 | Common variant |
| §Lower jaw prominent | 0 | 31 | ∞ (0.6-∞) | <0.001 | Common variant |
| §Dimpled/grooved chin | 1 | 27 | ∞ (0.5-∞) | <0.001 | Common variant |
| §Prominent ear | 8 | 40 | 5.5 (2.4-12.2) | <0.001 | Common variant |
| § ^a^ Ear lobes attached | 25 | 78 | 4 (2.4-6.9) | <0.001 | Common variant |
| §Clinodactyly (fingers) | 11 | 40 | 3.6 (1.8-7) | 0.001 | Common variant |
| §Clinodactyly (toes) | 9 | 48 | 5.8 (2.7-12.2) | <0.001 | Common variant |
| Inverted nipples | 6 | 28 | 4.6 (1.9-11) | 0.035 | Common variant |
| Toes widely spaced | 1 | 22 | 20 (2.9-∞) | 0.001 | Common variant |
| 2^nd^ toe longer than 1^st^ | 9 | 33 | 4.3 (1.9-9.8) | 0.021 | Common variant |
| §Pes planus | 4 | 36 | 8.8 (3.1-24.4) | <0.001 | Common variant |
| § ^a^ Sandal gap (toes) | 57 | 144 | 4.4 (2.8-6.8) | <0.001 | Common variant |
| §Dry skin | 12 | 60 | 6.9 (3.3-14.4) | <0.001 | Common variant |
| §Nails striped | 1 | 41 | 37.3(5.6-∞) | <0.001 | Common variant |
|  |  |  |  |  |  |

^a^ MPA in Waldrop scale

#Significant in girls (P < 0.05)

§Significant in boys (P <0.05)

Forty-eight morphological features occurring significant more frequently in patients with autism compared to controls in univariate analysis (McNemar’s test P <0.05) (Holm- Bonferroni corrected)
