# Supplementary material for: Predictive Value of Morphological Features in Patients with Autism versus Normal Controls
Source: J Autism Dev Disord. 2012 Jun 6;43(1):147–55. doi: 10.1007/s10803-012-1554-4 (PMC3536966; doi:10.1007/s10803-012-1554-4)
Supplement: Supplementary file 4 — Supplementary material 4 (DOC 28 kb) [file 10803_2012_1554_MOESM4_ESM.doc]

**Figure 1.** Terminology and Classification System of Morphological Abnormalities*

Morphological abnormality

Minor variant

(defect of phenogenesis)

Major abnormality

Malformation

(defect of embryogenesis)

Other abnormality

Deformation

Disruption

Dysplasia

Minor anomaly

(prevalence ≤ 4% in general population)

Common variant (prevalence > 4% in general population)

*Classification based on Merks et al. , 2003)
